# Cluster analysis of relapse risk factors in alcohol use disorder

**DOI:** 10.1192/j.eurpsy.2025.636

**Published:** 2025-08-26

**Authors:** J. Gajdics, O. Bagi, F. F. Farkas, B. K. Kádár, I. K. Pribék, B. Andó, B. A. Lázár

**Affiliations:** 1 Department of Psychiatry, University of Szeged, Albert Szent-Györgyi Medical School, Szeged, Hungary

## Abstract

**Introduction:**

The chronic relapsing nature of alcohol use disorder (AUD) makes treatment and recovery especially difficult. One of the most prevalent factors contributing to relapse is craving, which is a strong urge to drink alcohol. Moreover, alcohol withdrawal symptoms experienced during abstinence can trigger the urge to drink alcohol. Furthermore, stress and anxiety can also elicit craving and increase the motivation to drink alcohol.

**Objectives:**

Further understanding the risk of relapse would be crucial for the treatment of AUD. Thus, the aim of this study was to identify clusters within a sample of patients with AUD based on the different factors of relapse risk, and to compare these clusters based on the severity of alcohol use disorder, craving and anxiety.

**Methods:**

The sample consisted of 114 patients diagnosed with AUD at the Department of Psychiatry, University of Szeged, Hungary between November 2022 and January 2024. The level of AUD was measured with the Alcohol Use Disorders Identification Test (AUDIT) (subscales: consumption, dependence, harmful consequences of alcohol use) and the Severity of Alcohol Dependence Questionnaire (SAD-Q) (subscales: physical withdrawal signs, affective withdrawal signs, withdrawal relief drinking, quantity and frequency of alcohol consumption, rapidity of reinstatement of withdrawal symptoms following abstinence). State and trait anxiety were measured with the State-Trait Anxiety Inventory (STAI-S, STAI-T). Craving was measured with the Multidimensional Alcohol Craving Scale (MACS). The risk of relapse was measured with the Alcohol Relapse Risk Scale (ARRS) (subscales: stimulus-induced vulnerability (SV), emotionality problems (EP), compulsivity for alcohol (CA), negative expectancy for alcohol (NE), positive expectancy for alcohol (PE), insight into mental condition (IM)).

**Results:**

Two-step cluster analysis was performed with the six subscales of the ARRS as predictor variables. A two-cluster solution was found, in which SV proved to be the most important predictor. Independent sample t-tests for the two clusters revealed significant between-cluster differences on all subscales except for ‘lack of negative expectancy for alcohol’ (p ≥ 0.001). Independent sample t-tests and Chi-square tests were performed to compare the two clusters on the basis of age, sex, the severity of AUD, craving and anxiety. Significant differences were found in almost all factors except for age, sex and the ‘rapidity of reinstatement of withdrawal symptoms following abstinence’ subscale of the SAD-Q (p ≥ 0.01).

**Conclusions:**

The first cluster with more defined signs for the risk of relapse was characterised by more severe AUD, craving, state and trait anxiety compared to the second cluster with milder signs for the risk of relapse. These results suggest that the risk of relapse is a complex phenomenon, which can be identified through the evaluation of several different factors, which may influence treatment choices.

**Disclosure of Interest:**

None Declared

